# Comorbid Mood and Anxiety Disorder Diagnoses in Patients With First‐Episode Psychosis: Comparison Between Research and Clinical Diagnoses

**DOI:** 10.1111/eip.70239

**Published:** 2026-07-27

**Authors:** Maija Walta, Heikki Laurikainen, Reetta‐Liina Armio, Tiina From, Tero Vahlberg, Arvi Tolvanen, Raimo K. R. Salokangas, Jarmo Hietala

**Affiliations:** ^1^ Department of Psychiatry University of Turku Turku Finland; ^2^ Psychiatry Services, The Wellbeing Services County of Southwest Finland Turku University Hospital Turku Finland; ^3^ Department of Biostatistics Turku University and Turku University Hospital Turku Finland

**Keywords:** anxiety disorder, clinical care, clinical practice, depression, first‐episode psychosis, first‐episode schizophrenia, mood disorder

## Abstract

**Background:**

Clinically significant depression and anxiety disorders are common in different phases of first‐episode psychosis (FEP). These comorbidities can be undervalued in clinical practice. We studied how often comorbid depression and anxiety disorders are diagnosed using the SCID‐I interview in FEP patients and explored the corresponding clinical diagnoses for the same sample.

**Method:**

As a part of the Turku Early Psychosis Study (TEPS), we screened 3772 consecutive admissions during 5 years to the clinical psychiatric services of Turku Psychiatry. The final FEP sample in this study consisted of 94 patients with nonaffective (*n* = 67) and affective (*n* = 27) psychoses. Research diagnoses (SCID‐I) were compared with clinical diagnoses (ICD‐10) obtained from patient records 1 year from admission.

**Results:**

SCID‐I indicated that 42% (*n* = 28) and 9% (*n* = 6) of nonaffective FEP patients fulfilled the diagnostic criteria for lifetime (LT) and current (C) major depression. Depression diagnosis (F32 or F33) was recorded in only half of these cases in clinical practice during the 1‐year follow‐up. The SCID‐I also indicated that 45% (*n* = 42) and 48% (*n* = 45) of the whole FEP sample had LT and C anxiety disorders according to the SCID‐I. An anxiety disorder diagnosis (F40‐48) was recorded for only 40% of the patients with SCID‐based LT or C anxiety disorder in clinical practice during the 1‐year follow‐up period.

**Conclusion:**

Comorbid depression and anxiety disorders are common in FEP patients but are often not clinically diagnosed. A systematic diagnostic procedure in clinical practice will characterise the full syndromic nature of FEP for personalised treatment strategy.

## 1 Introduction

Comorbid depression and anxiety disorders are common in different stages of psychotic disorders. Upthegrove et al. (2010) estimated that as much as 80% of first‐episode psychosis patients experienced major depression during some part of the psychotic episode (Upthegrove et al. 2010). Other prevalence estimates of major depression suggested 7.5% in the 2 years prior to the onset of first‐episode psychosis, 6.9%–7.6% during the psychotic episode and 6.3% within 1 year of psychotic episode while the rates for anxiety disorder was reported to be 10%, 11%–12% and 8.8%, respectively (Strålin and Hetta 2021; Pope et al. 2013). It is also well established that individual anxiety disorders, such as social anxiety disorder and obsessive‐compulsive disorder, are common in first‐episode psychosis patients estimation prevalence varying 25%–32% for the social anxiety and 10.6%–13% for the obsessive‐compulsive disorder (Michail and Birchwood 2009; Voges and Addington 2005; Strakowski et al. 1995; Hagen et al. 2013). In the meta‐analysis of Wilson et al. (2020), depression and anxiety disorder co‐occurred at a similar rate in first‐episode psychosis, with 23% and 29% of FEP patients having depression and anxiety, respectively, even though the authors reported that prevalence studies were heterogeneous. Meta‐analysis was also based on symptom‐level estimation of the prevalence (Strålin and Hetta 2021; Pope et al. 2013; Wilson et al. 2020; Upthegrove et al. 2010; Michail and Birchwood 2009; Voges and Addington 2005; Strakowski et al. 1995; Hagen et al. 2013). To summarise, depression and anxiety symptoms and disorders are common in first‐episode psychosis patients, and the prevalence of such patients is considerably greater than that in the general population (Pirkola et al. 2005; World Health Organization 2017). However, these comorbidities may not be fully recognised in standard clinical practices with a focus on psychotic symptoms in early phases.

The importance of the detection of comorbidities is supported by the fact that depression and anxiety disorders affect the course of FEP. Depression in FEP patients increases the risk of suicidal thoughts, suicidal behaviour, suicide attempts, and suicide itself (Upthegrove et al. 2010; Ayesa‐Arriola et al. 2015; Bertelsen et al. 2007) and decreases quality of life (Sim et al. 2005; Mao et al. 2023) and functioning (McGinty and Upthegrove 2020). Like depression, anxiety symptoms and anxiety disorders in FEP patients are associated with a higher risk of suicidal ideation or attempts in these patients and poorer quality of life (Hagen et al. 2013; Ayesa‐Arriola et al. 2015; Sim et al. 2005; Mao et al. 2023), although the association with poor outcome in patients with first‐episode psychosis is not that clear (Karpov et al. 2021). Recently, Salokangas et al. (2025) reported that affective symptoms, anxiety and depression, at baseline and follow‐up, were linked to poorer functional outcomes in patients with early‐stage psychosis (Salokangas et al. 2025). Depression and anxiety disorders also affect recovery from symptoms of psychosis (Sim et al. 2006). While the prevalence of comorbid depression and anxiety disorders and their symptoms remain elevated in different stages of first‐episode psychosis, there is only little data how these comorbidities are taken into account in the treatment in clinical practice. Many studies have evaluated the diagnostic agreement between research diagnoses and clinician‐administered diagnoses of the primary psychiatric disorder (Davis et al. 2016). However, little is known about the diagnostic agreement of comorbidity diagnoses in first‐episode psychosis. Comorbidities often seem to be missed in typical clinical situations (Zimmerman and Mattia 1999) and Siu et al. (2018) reported that only approximately 40% of the comorbid disorders of FEP patients, such as depression, anxiety disorders, and substance disorders, discovered in the research setting in the SCID‐I were diagnosed by clinicians (Siu et al. 2018). However, the scarcity of studies investigating the detection of comorbidity diagnoses of FEP patients in clinical practice highlights the need for further studies to raise awareness of such comorbidities in the clinical care and treatment planning of FEP patients.

We here studied the Turku Early Psychosis Study (TEPS) sample to determine how often comorbid depression and anxiety disorders were identified in clinical practice among FEP patients. We then compared these results with those of the SCID‐I conducted with the same FEP patients in research settings. We have previously reported that this research FEP sample is approximately representative of the study population of FEP patients (Walta et al. 2022), which supports the generalisability of the study results to the clinical reality. We thus tested the hypothesis that the detection of comorbid depression and anxiety disorders differs between a research diagnostic setting and a clinical setting.

## 2 Methods

### 2.1 Study Group and Ethical Assessment

TEPS was approved by the Ethics Committee of the Hospital District of Southwest Finland in 2011 (approval number TEPS ETMK 64/180/2011). A detailed description of TEPS is available in a previous study (Salokangas et al. 2021). Briefly, TEPS is a clinical outcome study of early psychosis. In that study, patients were recruited from a single organisation, Turku Psychiatry, which became a part of the Turku University Hospital organisation in May 2017. Turku is a city with a population of more than 200 000 and is the sixth largest city in Finland.

All patient admissions in the units responsible for treating FEP in the Turku area (three inpatient units, one‐day hospital unit, and six outpatient units) were evaluated using eligibility questionnaires between October 2011 and December 2018. The eligibility questionnaire was completed by the clinical team to determine whether the patient had a psychotic episode or, alternatively, had a clinical high risk of psychosis with no previous psychotic episodes. Patients aged between 18 and 50 years were suitable for the study. According to the eligibility questionnaires, a total of 3772 patients were admitted to Turku Psychiatry between October 2011 and June 2016. All the questionnaires were evaluated by the research team to determine the patients eligible for inclusion in the study. Among these 3772 patients, 417 were preliminarily suitable to the study and of these 417 patients, 218 were willing to participate (Figure S1).

A total of 218 consenting patients were screened to determine whether they fulfilled the study's inclusion criteria. While TEPS study focused on primary psychosis disorders, TEPS exclusion criteria included presence of intellectual disability, a history of head trauma, presence of neurological brain disorder, and alcohol or substance dependence. Among the 218 patients, 37 patients either did not fulfil the inclusion criteria or dropped out of the study before the study procedure was initiated. Subsequently, 181 patients were interviewed using the Structured Clinical Interview for DSM‐IV diagnoses (SCID‐I/P) (First et al. 1997) and the Structural Interview of Prodromal Symptoms (SIPS) (Miller et al. 1999). According to the interviews, the patient group included 101 FEP patients, 40 patients who were at a clinically high risk for psychosis, and 40 patients who were classified into a separate risk patient control group with a clinically detected elevated risk for psychosis without fulfilling the SIPS/SOPS criteria. Finally, we included 101 patients with FEP in the study (Figure S1).

Two patients dropped out of clinical care before the SCID‐I/P was administered. We had to exclude these patients because of a lack of corresponding clinical information. Five patients dropped out of the study before the SCID‐I/P was administered. The final sample thus consisted of 94 FEP patients.

### 2.2 Hypothesis

We hypothesised that depression and anxiety disorders in FEP patients are underdiagnosed in clinical care. To explore this hypothesis, we considered the SCID‐I/P as the gold standard for diagnosing comorbid depression and anxiety disorders, against which we studied whether corresponding ICD‐10 diagnoses are detected in everyday clinical care. The term underdiagnosis is operationally defined as detected depression/anxiety disorder according to SCID‐I/P interview but no corresponding depression/anxiety disorder diagnosis (F32‐33, F40‐48) in clinical care during the 1‐year follow up. In this study, 1‐year follow‐up is defined as clinical follow‐up from the electronic patient records from the day the patient began the treatment for first‐episode psychosis in inpatient or outpatient care.

### 2.3 Data Collection

Electronic patient records were explored from the day the patient began treatment for first‐episode psychosis until the 1‐year follow‐up. All psychiatric inpatient and outpatient ICD‐10 diagnoses (F01‐99 Mental and behavioural disorders) were collected from the follow‐up period. We considered detection of depression in clinical care if the patient was set to F32 Single Depressive Episode or F33 Recurrent depressive disorder diagnosis, and we considered detection of anxiety disorder if the patient was set to F40 Phobic Anxiety Disorders, F41 Other anxiety disorders, F42 Obsessive Compulsive Disorders, F43 Reaction to severe stress and adjustment disorders, F44 Dissociative (conversion) disorders, F45 Somatoform Disorders, and F48 Other neurotic disorders diagnosis. Observation of clinical diagnoses was done retrospectively and independently from the clinical care, and therefore the clinician was not aware of the research study protocol. In this study, we chose clinical variables as independent variables to examine whether the patient was diagnosed with depression or an anxiety disorder in the clinical setting. These variables included sex, age, level of education, follow‐up time and number of visits to outpatient care.

In our study, SCID‐I/P interview covered both lifetime and current diagnoses. SCID‐I/P interview was performed after consent to participate was confirmed and treatment of first‐episode psychosis had begun (day 0 in the follow‐up). The protocol aimed to increase the reliability of the interview in a more stable state of the illness.

### 2.4 Statistical Analysis

The data were analysed using SPSS software (version 29.0). Differences in baseline variables between different patient groups were tested using the Pearson chi‐square test or Fisher's exact test. Normality was tested for continuous variables, and *t*‐tests or Wilcoxon rank tests were used according to the distribution. *p*‐Values less than 0.05 were considered statistically significant.

## 3 Results

### 3.1 Research Diagnoses (SCID‐I)

#### 3.1.1 Nonaffective and Affective Psychosis Samples

The mean interval between SCID‐I interview and the beginning of follow‐up was 58 days (range: 10–394 days). With one patient, there was an exceptional delay in performing the SCID‐I/P interview (394 d) time while the other 93 were interviewed close to the mean interval. The most common nonaffective psychosis diagnosis was the Psychosis NOS (23.4%, *n* = 22). For detailed information, see Table 1.

**TABLE 1:** SCID‐I‐based diagnoses; nonaffective and affective diagnoses; whole sample (*n* = 94).

	SCID (*n*, %)
Nonaffective FEP	
Psychosis NOS	22/94 (23.4%)
Schizophreniform psychosis	19/94 (20.2%)
Brief psychosis	7/94 (7.4%)
Delusional disorder	6/94 (6.4%)
Schizoaffective disorder	5/94 (5.3%)
Substance induced psychosis	4/94 (4.3%)
Schizophrenia	4/94 (4.3%)
Affective FEP	
Bipolar disorder	17/94 (18.1%)
Major depressive disorder with psychotic features	10/94 (10.6%)

#### 3.1.2 Mood Disorder (Depression) Diagnoses in Nonaffective Psychosis Sample (SCID)

Mood disorder diagnoses were studied only in a nonaffective psychosis patient sample. A total of 41.8% (*n* = 28) had a lifetime mood disorder, and 10.4% (*n* = 7) were suffering from a current mood disorder. For detailed information about other mood disorders, see Table 2. Mood disorders due to a general medical condition or substance‐induced disorder were not included.

**TABLE 2:** SCID‐I‐based mood disorder diagnoses among FEP patients; nonaffective group (*n* = 67).

	Lifetime % (*n*)	Current % (*n*)
Major depression disorder	26/67 (38.8%)	4/67 (6.0%)
Dysthymia	Nd^a^	1/67 (1.5%)
Mood disorder not otherwise specified	5/67 (7.4%)	3/67 (4.4%)
Any mood disorder	28 (41.8%)^b^	7 (10.4%)^c^

^a^ Not detected.

^b^ 4.5% (*n* = 3) of patients had major depressive disorder and mood disorder not otherwise specified.

^c^ 1.5% (*n* = 1) of patients had major depressive disorder and mood disorder not otherwise specified.

#### 3.1.3 Anxiety Disorder Diagnoses in First‐Episode Psychosis; Whole Study Sample (SCID)

A total of 44.7% (*n* = 42) of first‐episode psychosis patients had at least one lifetime anxiety disorder, and 21.3% (*n* = 20) had more than one lifetime anxiety disorder. A total of 47.9% (*n* = 45) of first‐episode psychosis patients had a current anxiety disorder. A total of 24.5% (*n* = 23) had more than one current anxiety disorder. For detailed information about anxiety disorders, see Table 3.

**TABLE 3:** SCID‐I‐based anxiety disorder diagnosis among FEP patients; whole study sample (*n* = 94).

Diagnosis	Lifetime (*n*, %)	Current *n* (%)
Panic disorder	19/94 (20.2%)	15/94 (16%)
Agoraphobia	6/94 (6.4%)	5/94 (5.3%)
Social phobia	17/94 (18.1%)	15/94 (16.0%)
Specific phobia	7/94 (7.4%)	6/94 (6.4%)
Obsessive compulsive disorder	6/94 (6.4%)	6/94 (6.4%)
Posttraumatic stress disorder	8/94 (8.5%)	6/94 (6.4%)
Generalised anxiety disorder		15/94 (16.0%)
Anxiety disorder NOS	10/94 (10.6%)	9/94 (9.6%)
Hypochondria		1/94 (1.1%)
Any anxiety disorder	42 (44.7%)^a^	45 (47.9%)^b^

^a^ 21.3% (*n* = 20) of patients had more than one lifetime anxiety disorder.

^b^ 24.5% (*n* = 23) of patients had more than one current anxiety disorder.

### 3.2 Clinical Diagnoses (ICD‐10)

#### 3.2.1 First Psychosis Diagnoses (During the First Hospital/First Outpatient Treatment Period)

A nonaffective psychosis diagnosis was made in 79.8% (*n* = 75) of the patients, and an affective psychosis diagnosis was made in 16.0% (*n* = 15) of the patients. A total of 4.3% (*n* = 4) patients did not receive a psychosis diagnosis. For detailed information, see Table 4.

**TABLE 4:** Clinical ICD‐10‐based baseline psychosis diagnosis in the whole FEP sample (*n* = 94).

	ICD‐10 (*n*, %)
Nonaffective FEP	
Nonspecified nonorganic psychosis (F29)	62/94 (66.0%)
Acute and transient psychotic disorder (F23)	7/94 (7.4%)
Persistent delusional disorder (F22)	4/94 (4.3%)
Schizoaffective disorder (F25)	1/94 (1.1%)
Schizotypal disorder (F21)	1/94 (1.1%)
Affective FEP	
Bipolar disorder (F31)	8/94 (8.5%)
Major depressive disorder with psychotic features (F32.3 or F33.3)	7/94 (7.4%)
No psychosis diagnosis	4/94 (4.3%)

#### 3.2.2 Psychosis Diagnoses by the Clinician at the End of Follow‐Up

The mean follow‐up period was 304 days from the beginning of the treatment. The follow‐up time ranged between 28 and 366 days (2016 was a leap year). At the end of the 1‐year follow‐up period, 43.6% (*n* = 41) of the patients were still diagnosed with F29 Unspecified Nonorganic Psychosis, 13.8% (*n* = 13) were diagnosed with F20 Schizophrenia, 5.3% (*n* = 5) were diagnosed with F25 Schizoaffective Disorder, 5.3% (*n* = 5) were diagnosed with F23 Acute and Transient Psychotic Disorder, 3.2% (*n* = 3) were diagnosed with F22 Persistent Delusional Disorder, and 1.1% (*n* = 1) were diagnosed with F21 Schizotypal Disorder. A total of 13.8% (*n* = 13) of the patients received an F31 Bipolar disorder diagnosis, and 9.6% (*n* = 9) received an F32.3 or F33.3 Major depressive disorder with psychotic features diagnosis. A total of 4.3% (*n* = 4) did not receive a psychosis diagnosis.

#### 3.2.3 F29 Diagnosis at Baseline and Diagnosis at the End of Follow‐Up

A total of 66% (*n* = 62) of the patients were initially diagnosed with F29. At the end of follow‐up, those patients were diagnosed as follows: F29 58.1% (*n* = 36), F20 14.5% (*n* = 9), F31 8.1% (*n* = 5), F25 6.5% (*n* = 4), F32.3 or F33.3 4.8% (*n* = 3), F23 4.8% (*n* = 3), F21 1.6% (*n* = 1), and F22 1.6% (*n* = 1).

### 3.3 Comorbid Diagnoses in Clinical Care Compared With That Made Using the SCID‐I

#### 3.3.1 Mood Disorders (Depression) in the Nonaffective Sample

To evaluate depression detection in clinical care, we combined the SCID‐I interview's lifetime major mood disorder and lifetime mood disorder not otherwise specified, together as one variable (lifetime depression), as well as the current major mood disorder and current mood disorder not otherwise specified as one variable (current depression). We did not include dysthymia, mood disorders due to a general medical condition, or substance‐induced mood disorders in this analysis.

A total of 41.8% (*n* = 28) of nonaffective FEP patients had experienced lifetime depression. A total of 53.6% (*n* = 15) of those who experienced lifetime depression were diagnosed with F32 or F33 during the follow‐up period in clinical care, and 46.4% (*n* = 13) did not receive F32 or F33 diagnoses in clinical care (Table 5). A statistically significant difference in sex, level of education, follow‐up time, or number of visits to clinical care was not found between these groups. Only age reached statistical significance (*p* = 0.04); patients who were diagnosed with depression in clinical care were younger (25.3 years) than those who were not diagnosed with depression (28.1 years) (Table S2).

**TABLE 5:** Mood disorders (depression) and anxiety disorders according to the SCID‐I and their diagnosis rates in clinical care during the 1‐year follow‐up period.

SCID‐I interview	ICD‐10 diagnoses *n*	ICD‐10 diagnoses %
Depression lifetime, *n* = 28	F32‐33, *n* = 15/28	F32‐33, 53.6%
Depression current, *n* = 6	F32‐33, *n* = 3/6	F32‐33, 50%
Anxiety disorder lifetime, *n* = 42	F40‐48, *n* = 16/42	F40‐48, 38.1%
Anxiety disorder current, *n* = 45	F40‐48, *n* = 16/45	F40‐48, 35.6%

Nine percent (*n* = 6) of nonaffective FEP patients had current depression according to the results of the SCID‐I. Fifty percent (*n* = 3) had F32 or F33 diagnoses during the follow‐up time in clinical care, and 50% (*n* = 3) did not have F32 or F33 diagnoses at all in clinical care (Table 5). The patient number was so small that a statistical comparison between these two groups was not possible.

#### 3.3.2 Anxiety Disorders in the Whole Sample

The full spectrum of anxiety disorders according to the SCID‐I and their detection rates in clinical care are described in Table S1.

To evaluate overall anxiety disorder detection in clinical care, we combined lifetime anxiety disorders, including panic disorder, agoraphobia, social phobia, specific phobia, obsessive compulsive disorder, posttraumatic stress disorder and anxiety disorder not otherwise specified, as one variable (lifetime anxiety disorder) and current anxiety disorders, including panic disorder, agoraphobia, social phobia, specific phobia, obsessive compulsive disorder, posttraumatic stress disorder, generalised anxiety disorder and anxiety disorder not otherwise specified, as one variable (current anxiety disorder). We did not include anxiety disorder due to general medical conditions or substance‐induced anxiety disorder in these variables. If the patient had more than one anxiety disorder, we calculated that as one incidence of lifetime or current anxiety disorder.

A total of 44.7% (*n* = 42) of all the FEP patients had one or more lifetime anxiety disorders according to the SCID‐I. A total of 38.1% (*n* = 16) of these patients received an F40‐48 diagnosis during the follow‐up time in clinical care, and 61.9% (*n* = 26) did not receive an F40‐48 diagnosis during the follow‐up time in clinical care (Table 5). A statistically significant difference in sex, age, level of education, follow‐up time, or number of visits to clinical care was not found between these groups (Table S3).

A total of 47.9% (*n* = 45) of the FEP patients were diagnosed with a current anxiety disorder according to the SCID‐I. A total of 35.6% (*n* = 16) of these patients were given an F40‐48 diagnosis during the follow‐up time by the clinician, and 64.4% (*n* = 29) were not given an F40‐48 diagnosis (Table 5). A statistically significant difference in sex, age, level of education, follow‐up time, or number of visits to clinical care was not found between these groups (Table S4).

## 4 Discussion

According to the results of the SCID‐I, nearly 50% of nonaffective FEP patients had a diagnosis of lifetime depression, and 10% had a current depression disorder. Similarly, over 40% of FEP patients suffered from a lifetime anxiety disorder, and over 40% currently suffered from it. The number of comorbidities in our sample was even higher than those suggested in previous comorbidity studies (Strålin and Hetta 2021; Pope et al. 2013; Wilson et al. 2020; Sim et al. 2006). However, this study confirms that a remarkable number of depressive and anxiety symptoms are present both before the onset and during the acute phase of first‐episode psychosis. Less than 50% of the patients diagnosed with depression or anxiety disorder according to the SCID‐I were diagnosed with depression or anxiety disorder in the clinical setting. The baseline demographics, follow‐up time, and number of visits to outpatient care did not have an association in this discrepancy.

Several factors may explain why detection rates in comorbid depression and anxiety disorder vary between research and clinical settings. First, the diagnostic procedures in the clinical setting differ in many ways from those in research setting, and studies have shown that diagnostic agreement varies considerably between these two settings (Taiminen et al. 2001; Ayano et al. 2021). In their meta‐analysis, Davis et al. (2016) established that anxiety disorders and substance use disorders tend to have lower diagnostic reliability, whereas schizophrenia seems to have higher diagnostic reliability (Davis et al. 2016). Therefore, the interrater reliability varies considerably between research and clinical settings, and our study also highlights this finding. Second, the disagreement may also be due to differences in how the research and clinical settings are constructed. Bhugra et al. (2011) studied the decision‐making of clinicians in psychiatry and reported that external factors such as time and the availability of treatment affect decision‐making, which might affect diagnostic procedures (Bhugra et al. 2011). Diagnoses in clinical settings are based on measures other than directly asking for symptoms from different diagnostic categories, such as interactions between patients and clinicians (Westen 1997). Third, the clinical setting, psychiatric diagnoses are used not only for clinical care but also for administrative purposes which may shift the focus from precise diagnostic procedure to more flexible and straightforward practice (Davis et al. 2016; First et al. 2018). As noted, many factors can explain why the detection rates of comorbidities in the research and clinical settings in our study varied. It seems that the procedure of making diagnoses differs between these two settings and that diagnostic specification is challenging in clinical care because of external factors such as time and treatment pressure and administrative purposes. In the era of rapid development of artificial Intelligence and machine learning procedures we may have tools for prediction of the risk for depression development in first‐episode psychosis in clinical care (Mena et al. 2025). Finally, the critical question is how underdiagnosis of depression and anxiety disorders affects treatment choices and outcomes in the patient of first‐episode psychosis. Such studies are the logical next step for this line of research with different study design and analysis of both biological treatments and psychosocial interventions.

While this study has focus on evaluation of comorbid mood and anxiety disorder in first‐episode psychosis, it is also notable that over half of those receiving primary psychosis diagnosis F29 Unspecified Nonorganic psychosis in clinical care at baseline are not diagnosed with precise psychosis disorder diagnosis during one‐year of follow‐up in clinical care. This is in line with register data in Finland indicating that F29 diagnosis is the most prevalent psychosis diagnosis in Finland (The Finnish National Quality Register for Psychosis Treatment 2025). Also, in the study of 2 years diagnostic stability of psychosis diagnosis, Psychosis NOS was still diagnosis with over 50% of patients receiving Psychosis NOS diagnosis in the baseline (Salvatore et al. 2009). Likewise, patient care relationships can affect the setting of the psychiatric diagnosis, that is, not using a schizophrenia diagnosis to avoid stigma for the patient or for relatives (Cohen et al. 2022). However, a delay in diagnosis may sometimes be necessary if further clinical characterisation is in progress (e.g., neuropsychology). However, in our view delaying precise diagnostics even beyond 1 year is problematic as diagnoses should provide a guidance for clinical treatment options chosen together with the patient. This finding together with our primary finding of underdiagnosis of affective disturbances in first‐episode psychosis emphasises the complex nature of defining specific psychosis diagnoses and it's comorbidities in early stages of these illnesses.

## 5 Limitations

Our study has several limitations. First, the classification systems compared in our study differ to some extent. The SCID‐I is based on the DSM‐IV classification, while the ICD‐10 is currently used in clinical settings in Finland. However, the diagnostic criteria for mood and anxiety disorders between these versions of the DSM and ICD classifications do not differ for the most part and are therefore comparable. Similarly, there are no major differences between the DSM‐IV and DSM‐V classifications with respect to anxiety and mood disorders. Second, we did not include patients with alcohol or substance dependence in our study, which excludes a notable patient group, and these results are not generalisable to this patient group. Third, our follow‐up time was only 1 year, which might affect these results, as diagnoses in psychiatry are specified within time. However, 1 year is a rather long period when considering patients' recovery and comorbidities affecting recovery.

## 6 Conclusion

Only 40%–50% of FEP patients for whom depression and anxiety diagnoses were made in the SCID‐I received these diagnoses in usual clinical care. Structural interviews and procedures may help clinicians detect the full spectrum of affective symptom dimensions in FEP patients in everyday clinical practice. Such procedures should be rigorously implemented, as they will be useful for clinical treatment planning and improving the long‐term functioning and quality of life of our patients.

## Funding

This work was supported by Turun Yliopistollinen Keskussairaala (P3848), Academy of Finland (278155, 323036), Suomen Kulttuurirahasto, Finnish Psychiatry Association, Turunmaan Duodecim seura, and Valtion tutkimusrahoitus.

## Conflicts of Interest

The authors declare no conflicts of interest.

## Supporting information


**Data S1:** Supporting Information.
**Figure S1:** Turku early psychosis study.
**Table S1:** Depression and anxiety disorders rate according to SCID‐I interview and corresponding diagnose rate in clinical care during 1 year of follow‐up.
**Table S2:** F32‐33 clinical diagnoses among non‐affective psychosis patients with lifetime mood disorder according to SICD‐I interview.
**Table S3:** F40‐48 clinical diagnoses among all first‐episode psychosis patients with lifetime anxiety disorder according to SICD‐I interview.
**Table S4:** F40‐48 clinical diagnoses among all first‐episode psychosis patients with current anxiety disorder according to SICD‐I interview.

## Data Availability

Research data are not shared.
