# Supplementary material for: Comorbid Mood and Anxiety Disorder Diagnoses in Patients With First‐Episode Psychosis: Comparison Between Research and Clinical Diagnoses
Source: Early Interv Psychiatry. 2026 Jul 27;20(8):e70239. doi: 10.1111/eip.70239 (PMC13406915; doi:10.1111/eip.70239)
Supplement: Supplementary file 1 — Data S1: Supporting Information. Figure S1: Turku early psychosis study. Table S1: Depression and anxiety disorders rate according to SCID‐I interview and corresponding diagnose rate in clinical care during 1 year of follow‐up. Table S2: F32‐33 clinical diagnoses among non‐affective psychosis patients with lifetime mood disorder according to SICD‐I interview. Table S3: F40‐48 clinical diagnoses among all first‐episode psychosis patients with lifetime anxiety disorder according to SICD‐I interview. Table S4: F40‐48 clinical diagnoses among all first‐episode psychosis patients with current anxiety disorder according to SICD‐I interview. [file EIP-20-0-s001.docx]

Supplement 1

Figure 1

Panic disorder

Lifetime panic disorder prevalence according to SICD-I interview was 20.2 % (n=19). 5.3 % (n=1) of those patients was set F40.0 diagnose during the follow up. Current panic disorder prevalence according to SICD-I interview was 16 % (n=15). 6.7 % (n=1) of those patients was set F40.0 diagnose during the follow up.

Among patients with lifetime panic disorder (n=19) in SCID-I interview, prevalence of other anxiety disorders than F41.0 according to ICD-10 were as following: F40.115.8 % (n=3), F41.1 10.5 % (n=2), F41.9 10.5 % (n=2) and F43.2 5.3 % (n=1). Among patients with current panic disorder (n=15) in SCID-I interview, prevalence of other anxiety disorders than F41.0 according to ICD-10 were as following: F40.1 20 % (n=3), F41.1 13.3 % (n=2), F41.9 % 13.3. % (n=2) and F43.2 6.7 % (n=1).

Agoraphobia

Lifetime agoraphobia prevalence according to SICD-I interview was 6.4 % (n=6). 33.3 % (n=2) of those patients was set F40.00 diagnose during the follow up. Current agoraphobia prevalence according to SICD-I interview was 5.3 % (n=5). 40 % (n=2) of those patients was set agoraphobia diagnose during the follow up time.

Lifetime and current agoraphobia did not had any other anxiety disorder diagnoses according to ICD-10 during the follow up time.

Social phobia

Lifetime social phobia prevalence according to SICD-I interview was 18.1 % (n=17). 29.4 % (n=5) of those patients was set F40.1 diagnose during the follow up. Current social phobia prevalence according to SICD-I interview was 16 % (n=15). 26.4 % (n=4) of those patients was set F40.1 diagnose during the follow up time.

Among patients with lifetime social phobia (n=17) in SCID-I interview, prevalence of other anxiety disorders than F40.1 according to ICD-10 were as following: F40.0 5.9 % (n=1), F41.1 11.8 % (n=2), F41.9 5.9 % (n=1), F42 5.9 % (n=1) and F45 5.9 % (n=1). Among patients with current social phobia (n=15) in SCID-I interview, prevalence of other anxiety disorders than F40.1 according to ICD-10 were as following: F40.0 6.7 % (n=1), F41.1 13.3 % (n=2), F41.9 6.7 % (n=1), F42 6.7 % (n=1) and f45 6.7 % (n=1).

Specific phobia

Lifetime specific phobia prevalence according to SICD-I interview was 7.4 % (n=7) and current specific phobia 6.4 % (n=6). There were no F40.2 diagnose during the follow up time.

Among patients with lifetime specific phobia (n=7) in SCID-I interview, prevalence of other anxiety disorders than F40.2 according to ICD-10 were as following: F41.8 14.3 % (n=1), F43.1. 14.3 % (n=1) and F44.9 14.3 % (n=1). Among patients with current specific phobia (n=7) in SCID-I interview, there were no other anxiety disorders than F40.2 according to ICD-10.

Obsessive compulsive disorder

Lifetime obsessive compulsive disorder prevalence according to SICD-I interview was 6.4 % (n=6). 16.7 % (n=1) was diagnosed F42 diagnose during the follow up. Current obsessive compulsive disorder prevalence according to SICD-I interview was 6.4 % (n=6). 16.7 % (n=1) of those patients was diagnosed with F42 during the follow up.

Among patients with lifetime obsessive compulsive disorder (n=6) in SCID-I interview, prevalence of other anxiety disorders than F42 according to ICD-10 were as following: F40.0 16.7 % (n=1). Among patients with current obsessive compulsive disorder (n=6) in SCID-I interview, prevalence of other anxiety disorders than F40.2 according to ICD-10 were as following: F40.0 16.7 % (n=1).

Posttraumatic stress disorder

Lifetime posttraumatic stress disorder prevalence according to SICD-I interview was 8.5 % (n=8). 25 % (n=2) of those patients was diagnosed F43.1 during the follow up time. Current posttraumatic stress disorder prevalence according to SICD-I interview was 6.4 % (n=6). 33.3 % (n=2) of those patients was diagnosed F43.1 during the follow up time.

Among patients with lifetime post-traumatic stress disorder (n=8) in SCID-I interview, prevalence of other anxiety disorders than F43.1 according to ICD-10 were as following: F40.1 12.5 % (n=1), F41.1 12.5 % (n=1), F41.8 12.5 % (n=1) and F44.9 12.5 % (n=1). Among patients with current post-traumatic stress disorder (n=6) in SCID-I interview, prevalence of other anxiety disorders than F43.1 according to ICD-10 were as following: F40.0 16.7 % (n=1), F41.1 16.7 % (n=1), F41.8 16.7 % (n=1) and F44.9 16.7 % (n=1).

Generalized anxiety disorder

Current generalized anxiety disorder prevalence according to SICD-I interview was 16 % (n=15). 6.7 % (n=1) was diagnosed anxiety disorder during the follow up time.

Among patients with current generalized anxiety disorder (n=15) in SCID-I interview, prevalence of other anxiety disorders than F41.1 according to ICD-10 were as following: F41.0 6.7 % (n=1), F41.9 6.7 % (n=1), F42 6.7 % (n=1), F43.1 6.7 % (n=1), F43.2 6.7 % (n=1) and F45 6.7 % (n=1).

Substance-induced anxiety disorder

Among patient with lifetime substance-induced anxiety disorder (n=1) there were no anxiety disorder according to ICD-10.

Anxiety disorder not otherwise specified

Lifetime anxiety disorder not otherwise specified prevalence according to SICD-I interview was 10.6 % (n=10). None of those patients was diagnosed F41.9. Current anxiety disorder not otherwise specified prevalence according to SICD-I interview was 9.6 % (n=9). None of those patients was diagnosed F41.9.

Among patients with lifetime not otherwise specified anxiety disorder (n=10) in SCID-I interview, prevalence of other anxiety disorders than F41.9 according to ICD-10 were as following: F40.00 10 % (n=1), F40.01 10 % (n=1) F40.1 20 % (n=2) and F42 10 % (n=1). Among patients with current not otherwise specified anxiety disorder (n=9) in SCID-1 interview, prevalence of other anxiety disorders than F41.9 according to ICD-10 were as following: F40.00 11.1 % (n=1), F40.01 11.1 % (n=2,), F40.1 11.1 % (n=1) and F42 11.1 % (n=1).

Hypochondria

Among patient with current hypochondria (n=1) in SCID-I interview, there were no other anxiety disorders according to ICD-10 during the follow up time.

Supplemental table 1. Depression and anxiety disorders rate according to SCID-I interview and corresponding diagnose rate in clinical care during 1 year of follow-up

| SCID based diagnosis (n) | Corresponding ICD-diagnosis set in clinical treatment during the follow-up and other anxiety disorders in specific anxiety disorder group % (n) |
| --- | --- |
| Depression |  |
| Major depression disorder and depression not otherwise specified LT (n=28) | F32 or F33  **53.6 % (n=15)** |
| Major depression disorder and depression not otherwise specified C (n=6) | F32 or F33  **50.0 % (n=3)** |
| Dysthymia C (n=1) | F34.1  **0 % (n=0)** |
| Anxiety disorders |  |
| Panic disorder LT (n=19) | **F41.0**  **5.3 % (n=1)**  F40.1 15.8 % (n=3)  F41.1 10.5 % (n=2)  F41.9 10.5 % (n=2)  F43.2 5.3 % (n=1) |
| Panic disorder C (n=15) | **F41.0**  **6.7 % (n=1)**  F40.1 20 % (n=3)  F41.1 13.3 % (n=2)  F41.9 13.3 % (n=2)  F43.2 5.3 % (n=1) |
| Agoraphobia LT (n=6) | **F40.0**  **33.3 % (n=2)**  no other anxiety disorder |
| Agoraphobia C (n=5) | **F40.0**  **40 % (n=2)**  no other anxiety disorder |
| Social phobia LT (n=17) | **F40.1**  **29.4 % (n=5)**  F40.0 5.9 % (n=1)  F41.1 11.8 % (n=2)  F41.9 5.9 % (n=1)  F42 5.9 % (n=1)  F45 5.9 % (n=1) |
| Social phobia C (n=15) | **F40.1**  **26.4 % (n=4)**  F40.0 6.7 % (n=1)  F41.1 13.3. % (n=2)  F41.9 6.7 % (n=1)  F42 6.7 % (n=1)  F45 6.7. % (n=1) |
| Specific phobia LT (n=7) | **F40.2**  **0 % (n=0)**  F41.8 14.3 % (n=1)  F43.1 14.3 % (n=1)  F44.9 14.3 % (n=1) |
| Specific phobia C (n=6) | **F40.2**  **0 % (n=0)**  no other anxiety disorder |
| Obsessive compulsive disorder LT (n=6) | **F42**  **16.7 % (n=1)**  F40.0 16.7 % (n=1) |
| Obsessive compulsive disorder C (n=6) | **F42**  **16.7 % (n=1)**  F40.0 16.7 % (n=1) |
| Posttraumatic stress disorder LT (n=8) | **F43.1**  **25 % (n=2)**  F40.1 12.5 % (n=1)  F41.1 12.5 % (n=1)  F41.2 12.5 % (n=1)  F41.8 12.5 % (n=1)  F44.9 12.5 % (n=1) |
| Posttraumatic stress disorder C (n=6) | **F43.1**  **33.3 % (n=2)**  F40.1 16.7 % (n=1)  F41.1 16.7 % (n=1)  F41.8 16.7% (n=1)  F44.9 16.7 % (n=1) |
| Generalized anxiety disorder C (n=15) | **F41.1**  **6.7 % (n=1)**  F41.0 6.7 % (n=1)  F41.9 6.7 % (n=1)  F42 6.7 % (n=1)  F43.1 6.7 % (n=1)  F43.2 6.7 % (n=1)  F45 6.7 % (n=1) |
| Anxiety disorder not otherwise specified LT (n=10) | **F41.9**  **0 % (n=0)**  F40.00 10 % (n=1)  F40.01 10 % (n=1)  F40.1 20 % (n=2)  F42 10 % (n=1) |
| Anxiety disorder not otherwise specified C (n=9) | **F41.9**  **0 % (n=0)**  F40.00 11.1 % (n=1)  F40.01 11.1 % (n=1)  F40.1 11.1 % (n=1)  F42 11.1 % (n=1) |
| Hypochondria C (n=1) | **F45.2**  **0 % (n=0)**  no other anxiety disorder |
| All anxiety disorders LT (n=43) ^†^ | F40-48, F19.8  38.1 % (n=16) |
| All anxiety disorders C (n=45) ^†^ | F40-48  35.6 % (n=16) |

LT=lifetime, C=current

† If patient had more than one anxiety disorder, it was calculated as one.

|  | F32-33 diagnose in clinical care (n=15) | No F32-33 diagnose in clinical care (n=13) | χ^2 (df)^ or Z | p-value |
| --- | --- | --- | --- | --- |
| Sex (%)  Women  Men | 33.3 % (n=5)  66.7 % (n=10) | 53.8 %(n=7)  46.2 % (n=6) | 1.20^(1)^ | 0.27 ^†^ |
| Age, median (IQR) | 23.0 (5.0) | 26.0 (8.0) | -2.04 | **0.04** ^‡^ |
| Level of education  Basic  Middle  High | 40.0 % (n=6)  60 % (n=9)  0 % (n=0) | 15.4 % (n=2)  61.5 % (n=8)  23.1 % (n=3) | 4.44^(2)^ | 0.11 ^§^ |
| Follow up time d, median (IQR) | 365.0 (50.0) | 365.0(273.0) | 0.63 | 0.56 ^‡^ |
| Number of visits in the clinical care,median (IQR) | 10.0(7.0) | 9.0 (19.0) | 0.51 | 0.62^‡^ |

Supplemental table 2. F32-33 clinical diagnoses among non-affective psychosis patients with lifetime mood disorder according to SICD-I interview

df = Degrees of freedom; IQR = Interquartile range

† Chi Square test

‡ Mann Whitney U test

§ Fisher’s exact test

Supplemental table 3. F40-48 clinical diagnoses among all first-episode psychosis patients with lifetime anxiety disorder according to SICD-I interview

|  | F40-48 diagnose in clinical care (n=16) | No F40-48 diagnose in clinical care (n=26) | χ^2 (df)^ or Z | p-value |
| --- | --- | --- | --- | --- |
| Sex (%)  Women  Men | 43.8 % (n=7)  56.3 & (n=9) | 50.0 % (n=13)  50.0 % (n=13) | 0.16 ^(1)^ | 0.69 ^†^ |
| Age,  median (IQR) | 25.0 (9.0) | 23.5 (8.0) | 0.34 | 0.74^‡^ |
| Level of education  Basic  Middle  High | 50.0 % (n=8)  31.3 % (n=5)  18.8 % (n=3) | 26.9 % (n=7)  46.2 % (n=12)  26.9 % (n=7) | 2.30^(2)^ | 0.32 ^†^ |
| Follow up time d, | 365.0 (1.0) | 365.0 (200.0) | 0.81 | 0.42^‡^ |
| median (IQR)  Number of visits in the clinical care  median (IQR) | 15.5 (17.0) | 11.5 (19.0) | 0.29 | 0.78^‡^ |

† Chi Square test

‡ Mann Whitney U test

Supplemental Table 4. F40-48 cllinical diagnoses among all first-episode psychosis patients with current anxiety disorder according to SICD-I interview

† Chi Square test

‡ Mann Whitney U test

§ Fisher’s exact test

|  | F40-48 diagnose in clinical care (n=16) | No F40-48 diagnose in clinical care (n=29) | χ^2 (df)^ or Z | p-value |
| --- | --- | --- | --- | --- |
| Sex (%)  Women  Men | 37.5 % (n=6)  62.5 % (n=10) | 51.7 % (n=15)  49.3 % (n=14) | 0.84^(1)^ | 0.36 ^†^ |
| Age,  median (IQR) | 26.0 (9.0) | 24.0 (8.0) | 0.76 | 0.45^‡^ |
| Level of education  Basic  Middle  High | 43.8 % (n=7)  37.5 % (n=6)  18.8 % (n=3) | 24.1 % (n=7)  55.2 % (n=16)  20.7 % (n=6) | 1.95^(2)^ | 0.43^§^ |
| Follow up time d  median (IQR) | 365.0 (1.0) | 365.0 (256.0) | 0.78 | 0.43^‡^ |
| Number of visits in the clinical care,  median (IQR) | 15.5 (17.0) | 9.0 (17.0) | 0.88 | 0.38^‡^ |
